# Galectin-3 Modulates Macrophage Activation and Contributes Smooth Muscle Cells Apoptosis in Abdominal Aortic Aneurysm Pathogenesis

**DOI:** 10.3390/ijms21218257

**Published:** 2020-11-04

**Authors:** Hsin-Ying Lu, Chun-Ming Shih, Chun-Yang Huang, Alexander T. H. Wu, Tsai-Mu Cheng, Fwu-Long Mi, Chun-Che Shih

**Affiliations:** 1 Division of Cardiovascular Surgery, Department of Surgery, Wan Fang Hospital, Taipei Medical University, Taipei 116, Taiwan; hsinying14@yahoo.com.tw; 2 Division of Cardiovascular Surgery, Department of Surgery, Taipei Veterans General Hospital, Taipei 112, Taiwan; chyhaung@hotmail.com; 3 Institute of Clinical Medicine, School of Medicine National Yang-Ming University, Taipei 112, Taiwan; 4 Taipei Heart Institute, Taipei Medical University, Taipei 110, Taiwan; cmshih53@tmu.edu.tw; 5 Division of Cardiology, Department of Internal Medicine, Taipei Medical University Hospital, Taipei 110, Taiwan; 6 The Ph.D. Program for Translational Medicine, College of Medical Science and Technology, Taipei Medical University, Taipei 110, Taiwan; chaw1211@tmu.edu.tw (A.T.H.W.); tmcheng@tmu.edu.tw (T.-M.C.); 7 Department of Biochemistry and Molecular Cell Biology, School of Medicine, College of Medicine, Taipei Medical University, Taipei 110, Taiwan; flmi530326@gmail.com; 8 Department of Surgery, School of Medicine, College of Medicine, Taipei Medical University, Taipei 110, Taiwan

**Keywords:** Galectin-3, abdominal aortic aneurysm, macrophage

## Abstract

Galectin-3 (Gal-3) is a 26-kDa lectin that regulates many aspects of inflammatory cell behavior. We assessed the hypothesis that increased levels of Gal-3 contribute to abdominal aortic aneurysm (AAA) progression by enhancing monocyte chemoattraction through macrophage activation. We analyzed the plasma levels of Gal-3 in 76 patients with AAA (AAA group) and 97 controls (CTL group) as well as in angiotensin II (Ang-II)-infused ApoE knockout mice. Additionally, conditioned media (CM) were used to polarize THP-1 monocyte to M1 macrophages with or without Gal-3 inhibition through small interfering RNA targeted deletion to investigate whether Gal-3 inhibition could attenuate macrophage-induced inflammation and smooth muscle cell (SMC) apoptosis. Our results showed a markedly increased expression of Gal-3 in the plasma and aorta in the AAA patients and experimental mice compared with the CTL group. An in vitro study demonstrated that the M1 cells exhibited increased Gal-3 expression. Gal-3 inhibition markedly decreased the quantity of macrophage-induced inflammatory regulators, including IL-8, TNF-α, and IL-1β, as well as messenger RNA expression and MMP-9 activity. Moreover, Gal-3-deficient CM weakened SMC apoptosis through Fas activation. These findings prove that Gal-3 may contribute to AAA progression by the activation of inflammatory macrophages, thereby promoting SMC apoptosis.

## 1. Introduction

An abdominal aortic aneurysm (AAA) is a common and potentially life-threatening degenerative vascular disease in the elderly population [1]. It is defined as local expansion of the abdominal aorta to greater than 50% of the normal diameter and is characterized by destruction of elastin and collagen in the media and adventitia, loss of smooth muscle cells (SMCs), with thinning of the medial wall, infiltration of lymphocytes and macrophages, and neovascularization [2]. Unclear pathological mechanisms have hampered the development of effective therapeutic strategies for AAAs, and surgical intervention is thus the only treatment option. However, recent evidence highlights that AAA pathogens play a major role in tissue-destructive inflammation that involves the accumulation of inflammatory cells in the adventitia through either recruitment of circulating monocytes or proliferation of resident macrophages [3]. Macrophages are present in several subsets with diverse functions. M1 macrophages mediate proinflammatory responses, whereas M2 macrophages exert anti-inflammatory effects and regulate wound healing [4]. M1 macrophages release and activate matrix metalloproteinases (MMPs), which can degrade the extracellular matrix and elastic laminae, thereby promoting lumen dilatation and thrombus formation [5].

Galectin-3 (Gal-3), a β-galactoside-binding lectin, is characterized by a conserved sequence within the carbohydrate recognition domain and amino-terminal tandem repeats [6]. At the cellular level, Gal-3 can be located in the cytosol and cell surface, and is sometimes translocated to the nucleus [7]. In the nucleus, Gal-3 regulates the transcription of proteins involved in cell adhesion, activation, proliferation, apoptosis, and migration by interfering with cAMP-response element binding protein (CREB) or the NF-κB transcription factor [8]. Gal-3 also serves as a proinflammatory mediator and has been associated with several pathologies, many of which encompass various inflammatory responses, heart diseases, cancers and systemic sclerosis [8,9,10]. Gal-3 has recently been reported as a prognostic marker for cardiovascular disease. Several studies have suggested that Gal-3 is an emerging biomarker linked to myocardial fibrosis, tissue remodeling, and heart failure development; it has also been shown to be associated with heart failure severity [11,12,13]. Levels of plasma Gal-3 were markedly high in patients with coronary artery disease [14]. In addition, Fernandez-García CE et al. suggest that increased Gal-3 levels are associated with AAA involving in CCL5 expression by STAT3 activation [15]. The present study identified that Gal-3 serves as novel contributor in AAA progression, by promoting macrophage-driven aortic inflammation and smooth muscle cell apoptosis.

## 2. Results

### 2.1. Increased Expression of Gal-3 in AAA

Total of 76 AAA patients and 97 control subjects were enrolled in this study. The clinical and laboratory features of the subjects were shown in Table 1.

**Figure 1:**
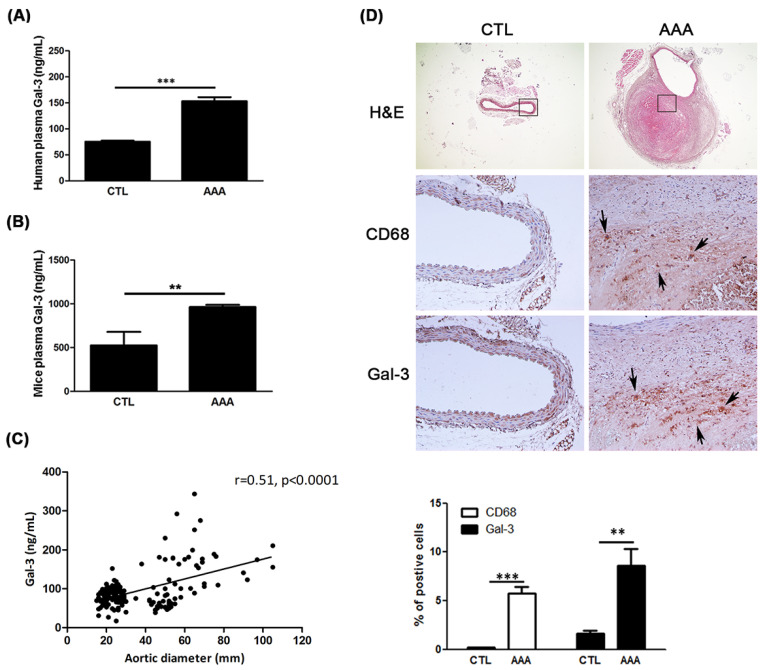
Increased expression of Gal-3 in patients and mice with AAA. (**A**,**B**) A marked increase in Gal-3 concentration in the plasma was observed in patients (CTL group, *n* = 97, AAA group, *n* = 76) and mice (*n* = 5 each group) with AAA compared with CTL. Data are expressed as mean ± S.E.M. ** *p* <0.01, *** *p* < 0.0001. (**C**) The correlation between aortic diameter and plasma levels of Gal-3. (**D**) Representative aortic sections of mice stained with H&E, Gal-3 and CD68. The black arrow indicated macrophage and Gal-3. A significantly increased macrophages infiltration as well as Gal-3 expression was found in the aneurysmal aorta. The magnification of immunostaining images is 200×.

To determine whether plasma Gal-3 levels are associated with AAAs, the plasma level of Gal-3 in human and mice was measured using ELISA. As shown in Figure 1A,B, the plasma level of Gal-3 was markedly higher in the AAA group than it was in the CTL group. Additionally, the Spearman correlation between aortic diameter and Gal-3 was significant (*r* = 0.51, *p* < 0.0001) as shown in Figure 1C. The logistic regression was used to demonstrate the diagnostic value of increased Gal-3 plasma concentrations for AAA with and without clinical variables’ available. Model 1 was adjusted for demographic characteristics (age, sex). Model 2 was adjusted for clinical characteristics (hyperlipidemia, diabetes mellitus (DM), chronic obstruction pulmonary disease (COPD). Model 4 was adjusted for the use of drugs. Collectively, the results in Table 2 indicated that the higher level of Gal-3 was associated with incident AAA.

To evaluate the pathophysiological pattern of Gal-3 and macrophage in AAAs, aneurysmal specimens from experimental mice were immuno-stained with of Gal-3 and CD68. AAA mice showed increased expression of Gal-3 and CD68 compared with the CTL mice (Figure 1D). The increased expressions of Gal-3 were related to macrophage infiltration in aorta. Furthermore, plasma Gal-3 concentration significantly increased in AAA mice compared with CTL mice. These observations suggest that Gal-3 may play a modulator in AAA.

### 2.2. Gal-3 Modulated Inflammatory Regulators in Macrophages

**Figure 2:**
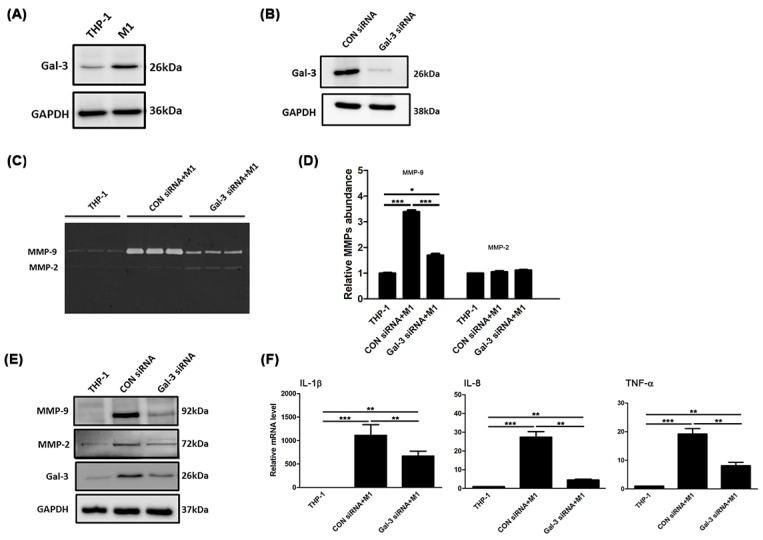
Gal-3 was involved in the modulation of inflammatory regulators in.macrophages. (**A**) THP-1 monocytes were stimulated with LPS and IFN-γ that were considered M1 macrophages. Gal-3 expression was analyzed using Western blots analysis. GAPDH was used as a loading control. The M1 cells exhibited increased Gal-3 expression. (**B**) Western blots analysis was used to detect the effect of Gal-3 expression knockdown by siRNA in M1 cells. (**C**) Gelatin zymography detected MMPs activity. The M1 macrophages markedly increased MMP-9 activity, which could be inhibited by Gal-3 blockade. (**D**) Histogram representing the quantified proteolytic activity. (**E**) The protein expression of MMP-9, MMP-2 and Gal-3 was confirmed by Western blots analysis. (**F**) Quantitative RT-PCR was performed to analyze IL-8, TNF-α, and IL-1β mRNA expression. Data are expressed as mean ± S.E.M. for at least 3-4 independent experiments. * *p* <0.05, ** *p* < 0.001, *** *p* < 0.0001.

AAA expansion is characterized by vascular inflammation and proteolysis. Therefore, to investigate whether Gal-3 is altered by monocyte-macrophage differentiation, THP-1 monocytes were used to polarize to M1 macrophage. As shown in Figure 2A, the M1 macrophages showed a significant increase in Gal-3 expression compared with THP-1 cells. Next, siRNA-based silencing was used to investigate the role of Gal-3 in M1-mediated inflammation and proteolysis. Specific targeting of Gal-3 with commercially available galectin siRNA led to a marked reduction in the expression of Gal-3 in M1 macrophages, as shown using Western blot analysis to assess the efficiency of the transfection process (Figure 2B). We first examined the MMP-9 and MMP-2 abundance by using zymography. The result showed that the increased MMP-9 abundance in macrophages was eliminated by Gal-3 inhibition, but not MMP-2 (Figure 2C,D). In Figure 2E, knockdown Gal-3 reversed the expression of MMP-9 and MMP-2 in M1 cells. The inflammatory genes in M1 cells formed in response to Gal-3 siRNA treatment were recognized by real-time RT-PCR. Blocking Gal-3 expression decreased IL-8, TNF-α and IL-1β messenger RNA expression in M1 macrophages (Figure 2F). These results suggest that a declining level of Gal-3 attenuates macrophage-associated inflammatory cytokine expression.

### 2.3. Gal-3 Promoted Up-Regulation of MCP-1 to Enhance the Chemotaxis of Monocytes

**Figure 3:**
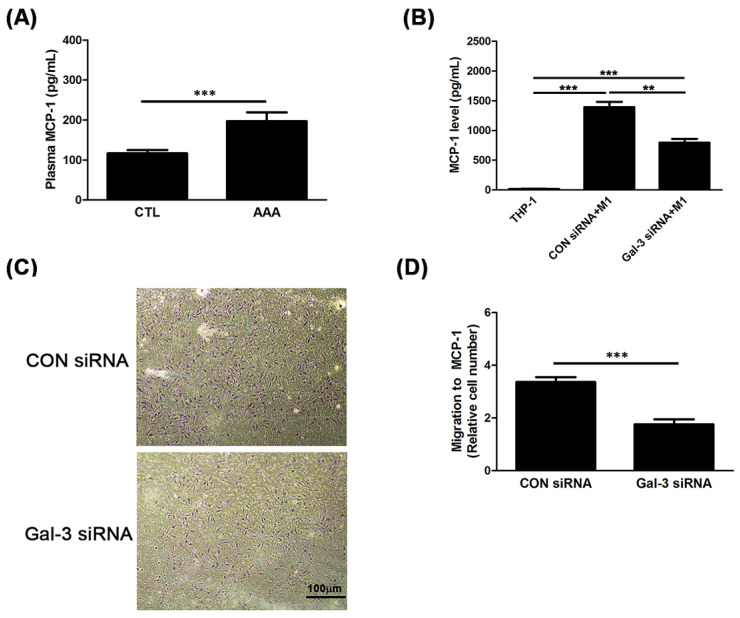
Gal-3 enhanced monocyte chemotaxis through of MCP-1 up-regulation. (**A**,**B**) ELISA was used for detection of MCP-1 concentration in human plasma and CM. (**C**,**D**) Representative images of transwell migration assays in response to MCP-1. M1 cells were stained with crystal violet and counted under microscope. The magnification was 100x. Data are expressed as mean ± S.E.M. for at least 3–4 independent experiments. ** *p* < 0.001, *** *p* < 0.0001.

Monocyte chemoattractant protein (MCP-1) regulates macrophage in AAA. The plasma level of MCP-1 in human was explored. As shown in Figure 3A, the plasma level of MCP-1 was markedly higher in the AAA group than it was in the CTL group (136.2 ± 122.3 pg/mL vs. 222.1 ± 143.7 pg/mL; *p* < 0.0001). To further elucidate whether Gal-3 regulate macrophage migration through MCP-1 action, we examined the concentration of MCP-1 in CM, and chemotaxis assay was performed using transwell assay. Our data demonstrated that the increased MCP-1 level in M1 CM was reduced by Gal-3 inhibition (Figure 3B). Additionally, compared with cells transfected with CON siRNA, Gal-3 siRNA transfected cells showed a significant decrease in the number ofmigrated cells to the media containing with MCP-1 (Figure 3C,D). The results demonstrated that Gal-3 enhances macrophages motility through MCP-1.

### 2.4. Inhibited Gal-3 Attenuated Macrophage-Induced Death of SMCs

**Figure 4:**
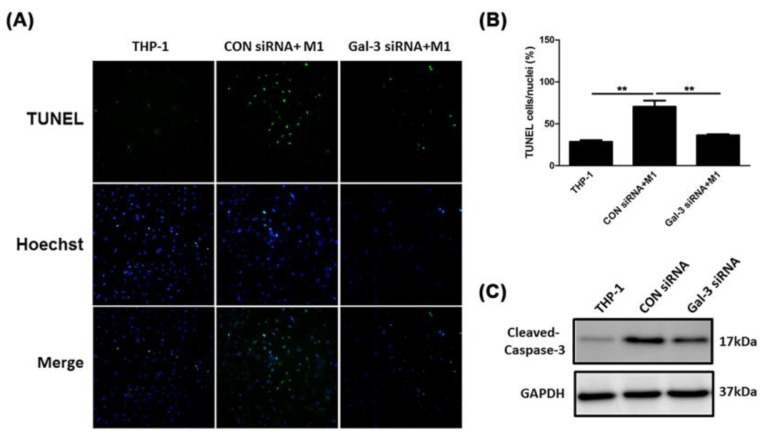
Inhibition of Gal-3 attenuated macrophage-induced death of SMCs. A7R5 cells were incubated with different treatment CM, respectively. (**A**) Representative photographs of TUNEL staining. Apoptotic cells are shown in green and cell nuclei are shown in blue. The magnification was 200×. (**B**) Quantitative analysis of apoptotic cell death was represented as the percentage of TUNEL positive nuclei. ** *p* < 0.001. (**C**) Representative Western blots demonstrating expression in the active, cleaved form of caspase-3 protein; GAPDH was used as a loading control.

Reduced smooth muscle cell (SMC) density within the elastic media of a human aneurysmal wall has been found to be associated with increased SMC apoptosis by inducing macrophage infiltration [16,17]. To determine whether Gal-3 in macrophages affects SMC apoptosis, A7R5 aortic SMCs were incubated with M1 macrophage CM of M1 cells that were transfected with Gal-3 siRNA or CON siRNA. TUNEL staining was performed to evaluate the cell apoptosis. The results are presented in Figure 4A, where the green cells represent apoptotic cells. Compared with the THP-1 CM group, incubation with M1 CM resulted in an increase in apoptotic cells, whereas a lower quantity of positive cells was observed after inhibition of Gal-3 with M1CM-incubated SMCs (Figure 4B). In addition, M1 CM-incubated SMCs elevated the expression of cleaved caspase-3; moreover, the effects of M1 CM induction were weakened after Gal-3 knockdown (Figure 4C). These results demonstrate that inhibition of Gal-3 led to attenuate SMCs from M1 CM-induced apoptosis.

### 2.5. Resistance of Gal-3 to Macrophage-Induced SMC Apoptosis Associated With Fas Deactivation

**Figure 5:**
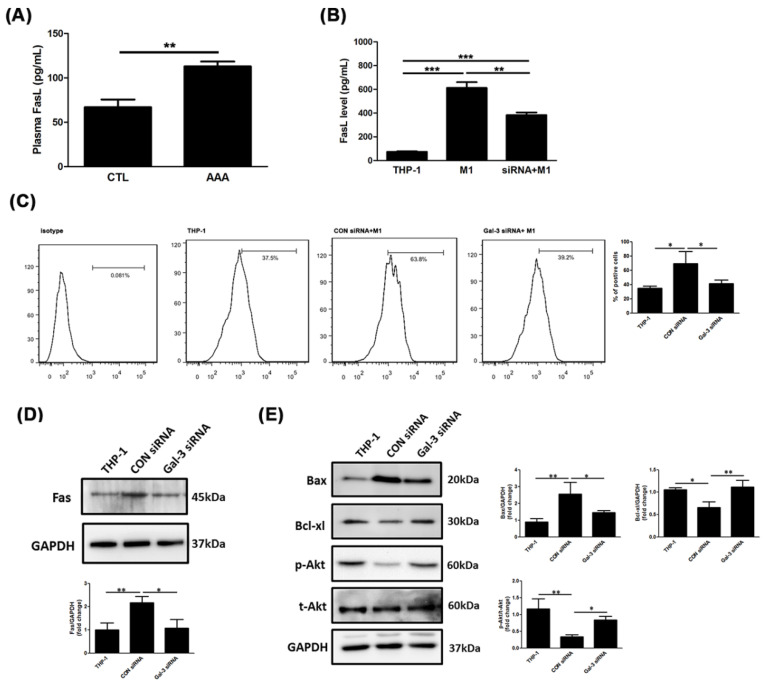
Gal-3 blockade attenuated macrophage-induced SMC apoptosis by Fas deactivation. (**A**,**B**) Comparison of soluble FasL level in human plasma and CM. Data are expressed as mean ± S.E.M. * *p* <0.05, ** *p* < 0.001, *** *p* < 0.0001. (**C**) Expression of Fas a surface death receptor on A7R5 cells was evaluated using flow cytometry analysis. All experiments were repeated independently in triplicate. (**D**,**E**) Expression levels of Fas, Bax, and Bcl-xl and phosphorylation of Akt and total Akt were analyzed through Western blots analysis; GAPDH was used as a loading control. All experiments were repeated independently in triplicate.

Expression level of apoptosis-inducing ligands and receptors was previously reported to induce cell death and caspase activation. Evidence suggests that macrophages must express FasL on the cell surface and SMCs must express Fas on the cell surface [18]. As shown in Figure 5A explored to soluble FasL in human plasma showed a higher level in the AAA group than it was in the CTL group (73.7 ± 34.2 pg/mL vs. 95.8 ± 44.6 pg/mL; *p* < 0.0001). To demonstrate whether the Gal-3 affects macrophage-induced SMC apoptosis through Fas, the level of FasL in CM was exanimated. Treatment with Gal-3 siRNA markedly decreased M1-induced soluble FasL level (Figure 5B). Next, the expression of Fas in M1-incubated SMCs was assessed with or without Gal-3 siRNA transfection using flow cytometry. The expression intensity of Fas in SMCs after M1 CM stimulation was higher than that in the THP-1 CM incubation, but the intensity of Fas was weakened by Gal-3 siRNA + M1 CM treatment (Figure 5C). Western blotting confirmed the same result as shown in Figure 5D.

We then examined the mechanism underlying the effect of Gal-3 inhibition on CM-induced cell death. In addition, several apoptosis-related proteins were examined using Western blot analysis, including Bax, Bcl-xl, and Akt. As shown in Figure 5E, the results revealed that Gal-3 inhibition decreased M1 CM-induced Bax expression, whereas the production of antiapoptotic proteins and cell-survival-promoting molecules such as Bcl-xl and Akt were upregulated. The results indicate that the inhibition of Gal-3 in macrophages was highly effective in rescuing SMCs from death.

## 3. Discussion

In recent years, the potential role of Gal-3 as a circulating biomarker of cardiovascular diseases has been supported by several studies. Our findings indicated that plasma Gal-3 levels were significantly higher in the AAA group than they were in the CTL group because of a strong association with macrophage activation, at least in part, by increasing MPC-1 level. A decline in Gal-3 deactivates macrophage-induced SMCs apoptosis through downregulated inflammation and Fas expression. This suggests that Gal-3 may be a novel apoptotic regulator in response to inflammatory macrophages activation in AAAs.

Several previous studies have identified a strong clinical association between smoking and AAA development [19,20,21]. Besides smoking, other risk factors include male sex, age, hypertension, COPD, hyperlipidemia, and a family history of the disorder [20,22]. The results of a large population-based cohort agree with our study, where it demonstrated that the plasma Gal-3 concentration was positively associated with AAA incidence. However, the association does not vary by age, race, or sex, and exhibits independently of demographic characteristics and several AAA risk factors. [23]. Whether the plasma Gal-3 level might be pharmacologically changed is unknown.

Gal-3 is a versatile protein that orchestrates several physiological and pathophysiological processes in the cardiovascular disease. It relates to the inflammatory cascade following cardiac injury and to pathways regulating cardiac contractility [24]. Moreover, plasma Gal-3 can predict cardiovascular death in high-risk patients with coronary angiography [25]. Gal-3 additionally can be found in foam cells of atherosclerotic animal models [26], and deactivation of Gal-3 gene expression markedly reduces the progression of atherosclerosis [27]. Plaque foam cells secrete Gal-3, which attracts monocytes and macrophages, thereby further stimulating the progression of atherosclerosis [28]. Fernandez-García CE et al. show an association between plasma Gal-3 levels and AAA progression [15]; this finding is consistent with the results of our experiment. After adjustment for cofounding factors as demonstrated in our analyses, Gal-3 levels remained significantly higher among participants with AAA compared to participants without AAA. We notably demonstrate that Gal-3 involves in macrophage activation and mediate SMCs apoptosis reflecting to AAA formation.

MMPs contribute to inflammatory processes depending on the cell type and disease state. Evidences have shown a critical role for some MMPs in AAA pathogenesis [29]. Targeted gene disruption of MMP-2 and MMP-9 suppresses development of experimental AAAs [30]. Gong Y et al. indicates that MMP-9 activates inflammatory macrophages migration and progression of AAA [31]. We have now demonstrated that Gal-3 knockdown in M1 abrogates MMP-9 levels. Circulating inflammatory cytokines have been observed to be elevated in patients with a variety of inflammatory diseases. Previous research revealed an increase ininfiltrated macrophages exhibiting a robust production of cytokines, including IL-6, IL-8, TNFα, and IL-1β, and specific patterns of macrophage polarization involving in aortic inflammation and aneurysm formation [32]. Given the role of macrophages in diverse disease pathologies at every stages of the inflammatory process, they may provide a novel approach to therapy. Gal-3 has been reported to be involved in many processes during acute inflammatory responses, including neutrophil activation and adhesion [33], chemoattraction of macrophages [28], and activation of mast cells [34]. We have found that monocyte-macrophage differentiation amplifies inflammation by inducing the expression of a series of well-known pro-inflammatory molecules. This effect is weakened by Gal-3 reduction, indicating that Gal-3 is responsible for macrophage activation.

Histological examinations of AAAs in humans and animals have revealed a paucity of medial vascular SMCs in these specimens, which are associated with the SMC apoptosis [2]. Numerous studies have indicated that activated monocytes and macrophages release several primarily proapoptotic cytokines and death ligands such as soluble FasL to induce SMC apoptosis directly in other cells [35]. Gal-3 is reportedly involved in apoptosis. Intracellular Gal-3 can inhibit apoptosis [36], whereas extracellular Gal-3 induces T-cell apoptosis [37]. The current study demonstrated that CM from macrophages with Gal-3 blockade can attenuate the death of SMCs with respect to Fas regulation. 

## 4. Materials and Methods 

### 4.1. Patient Recruitment and Blood Sample Collection

We studied 76 patients with AAA who visited the AAA clinic in the Taipei Veterans General Hospital and verified aortic dimeter greater than 3 cm using computed tomography and vascular surgical evaluation as AAA group. The 97 subjects without aortic disease as control group (CTL group) were included from patients regularly visiting the cardiology clinic. Baseline demographic data, functional status, cardiovascular risk factors and medication were also recorded in Table 1. Age, sex, hypercholesterolemia, diabetes mellitus, and chronic obstruction pulmonary disease (COPD) differed significantly between the CTL group and AAA group. The use of angiotensin-converting enzyme inhibitors (ACEi), angiotensin receptor blocker (ARB), statins, and antiplatelet also differed significantly between the CTL group and AAA group. Patients with negative histories of heart failure, neoplastic, rheumatological, and immunological diseases were included. The ethics committee in charge of research on humans at the Taipei Veterans General Hospital granted approval for this study (approval number 2016-07-013AC, approval date: 26 July 2016). Moreover, all participating patients signed consent forms to participate in this study. Peripheral blood was collected from all participants in ethylene-diamine-tetra-acetic acid tubes (EDTA). All samples were processed within 3 h following collection. The blood samples were centrifuged (3000× *g*, 10 min, 4 °C) to remove cells and debris. The supernatants were transferred to ribonuclease-free tubes and stored at −80 °C until required.

### 4.2. Enzyme-Linked Immunosorbent Assay

The enzyme-linked immunosorbent assay (ELISA) was performed according to the manufacturer’s protocol to determine the plasma concentrations of Gal-3 in human (R&D Systems, Minneapolis, MN, USA) and experimental mice (RayBiotech, Norcross GA, USA). The absorbance of each sample was determined using a microplate reader (Infinite M1000, TECAN, Austria, Europe).

### 4.3. Animal Aortic Collection

Male apoE-/- mice on a C57BL/6 background were purchased from Jackson Laboratories (Bar Harbor, ME, USA). The animal studies were approved by the Institutional Animal Care and Use Committee of the Taipei Veterans General Hospital (Taipei, Taiwan). All animal experiments were conducted at the Taipei Veterans General Hospital. Mice were kept in micro-isolator cages on a 12-h day/night cycle. Water and a normal laboratory diet were available ad libitum. All experiments were performed in 12- to 14-week-old male apoE-/- mice. Mice were infused subcutaneously with Ang II (1000 ng/kg/min; Sigma, St. Louis, MO, USA) (AAA group) or normal saline (CTL group) via mini-osmotic pumps (model 2004, Alzet, Palo Alto, CA, USA) for 28 days. Mice were sacrificed under anesthesia by intraperitoneal injection of one dose of Zoletil 50 (10 mg/kg) and Xylazine (10 mg/kg). The aorta tissue and blood were collected for further analysis.

### 4.4. Histological and Immunohistochemistical Staining

Tissues were dehydrated through a graded ethanol series, cleared with xylene, infiltrated with warm paraffin, embedded in paraffin blocks, and cut to a 4-μm thickness. To evaluate the morphology, hematoxylin/eosin (H&E) staining was performed. For immunohistochemistry, cross sections were treated with 0.01 M sodium citrate buffer (PH 6.0) by a microwave-based antigen retrieval technique for 20 min, then 3% H_2_O_2_ for 10 min to block endogenous peroxidase activity. Anti-CD68 and anti-Gal-3 (Abcam, Cambridge, MA, USA) were incubated for 24 h at 4 °C and secondary antibodies for 1 h at room temperature. After staining with DAB, the slides were visualized with microscope.

### 4.5. RNA Interference of Gal-3 Using Small-Interfering RNA

Knockdown of Gal-3 gene expression was performed via the transfection of cells with small-interfering RNA (siRNA) using Lipofectamine RNAiMax (Invitrogen, Carlsbad, CA, USA). 2 × 10^5^ cells were suspended in the transfection medium containing the transfection reagent and 30 nM human Gal-3 siRNA (Gal-3 siRNA) duplex mixtures or control siRNA (CON siRNA) (GE Healthcare, Piscataway, NJ, USA).

### 4.6. Cell Lines and Cell Culture

THP-1 cells, constituting a human leukemic cell line, were obtained from the American Type Culture Collection (ATCC, Manassas, VA, USA) and grown in RPMI 1640 medium with 2 mM l-glutamate, 4.5 g/L glucose, 10 mmol/L HEPES, 1.0 mmol/L sodium pyruvate, 10% fetal bovine serum (FBS), and 1% antibiotic antimycotic mixture. Cell density was maintained between 5 × 10^4^ and 8 × 10^5^ viable cells/mL, and the medium was refreshed every 2–3 days. THP-1 monocytes were differentiated into macrophages through 24 h incubation with 100 nM phorbol 12-myristate 13-acetate (PMA) (Sigma, St. Louis, MO, USA). Macrophages were polarized to M1 macrophages through addition of 100 ng/mL lipopolysaccharide (LPS) (Sigma-Aldrich) and 20 ng/mL Interferon gamma (IFN-γ) (R&D Systems). Cells were then washed with PBS twice and cultured with RPMI 1640–1% FBS for 48 h. The conditioned media (CM) were harvested and filtered through a 0.22-μm filter to remove cell debris to obtain THP-1 CM, CON siRNA + M1 CM and Gal-3 siRNA + M1 CM, and stored in aliquots at −20 °C. The A7r5 cells, the rat aortic SMCs, were purchased from the ATCC and were expanded in DMEM supplemented with 2 mM glutamine and 10% FBS at 37 °C in an incubator perfused with 5% CO2 and 95% O2. Prior to experimentation, the A7r5 cells were exposed to CM for the subsequent studies.

### 4.7. Chemotaxis Assay

M1 macrophages following CON or Gal-3 siRNA transfection were detached using trypsin. 2 × 104 of cells in 100 μL of serum free RPMI 1640 were placed above the inserts, and the lower chamber contained 700 μL of RPMI1640 medium with monocyte chemoattractant protein-1 (MCP-1). Following incubation at 37 °C for 6 h, cells on the upper side of the membrane were removed using clean swabs and cells on the lower side were fixed with 70% cold ethanol and then stained with 0.2% crystal violet solution. After washing with PBS three times, migrated cells were observed using the microscopy and counted five high power fields in triplicate.

### 4.8. Gelatin Zymography

As previously described, gelatin zymography was used to determine the gelatinolytic activity of MMP-2 and MMP-9 in cell culture supernatants as previously described. Experiments were performed in triplicate. Briefly, equivalent amounts of sample were electrophoresed under non-reducing conditions on 7.5% sodium dodecyl sulfate-polyacrylamide gel electrophoresis (SDS-PAGE) gels containing 0.1 mg/mL gelatin as a substrate. The gels were washed in a buffer solution containing 2.5% Triton X-100 for 1 h to remove SDS, and they were incubated with a substrate buffer at 37 °C for 18 h.

### 4.9. Quantitative Real-Time Polymerase Chain Reaction

Total RNA was isolated using an RNeasy Mini Kit (Invitrogen) according to the manufacturer’s instructions. The RNA concentration and purity were determined using standard spectrophotometric methods. Subsequently, 1 μg of RNA sample was used for reverse transcription and the synthesis of complementary DNA by using the Superscript III First-Strand system (Invitrogen). Quantitative real-time polymerase chain reaction (RT- PCR) was performed using a FastStart DNA Master SYBR Green I kit and a LightCycler (Roche, CA, USA). For quantification of gene expression changes using a ABI StepOne Real-Time PCR System (ABI, CA, USA), the ΔΔCt method was used to calculate relative fold changes normalized to GAPDH. RT-PCR primers for specific target genes were designed based on their reported sequences and synthesized by the Genomics BioSci & Tech Co., Ltd. (Taipei, Taiwan) as follows (5′−3′): IL-8, sense: TCC TGA TTT CTG CAG CTC TGT, antisense: AAA TTT GGG GTG GAA AGG TT; TNF-α, sense: ATG AGC ACA GAA AGC ATG ATC, antisense: TAC AGG CTT GTC ACT CGA ATT; IL-1β, sense: GAC CTT CCA GGA TGA GGA CA, antisense: AGG CCA CAG GTA TTTTGT CG; GAPDH, sense: TCA CCA CCA TGG AGA AGG C, antisense: GTC AAG CAG TTG GTG GTG CA.

### 4.10. Western Blotting

Cells were washed with buffer containing 150 mM NaCl, 50 mM Tris–HCl pH 7.4, 1% NP-40, and inhibitors for phosphatase and proteinase (Roach). Lysed cells were put on ice for 30 min and centrifuged at 10,000× *g* for 30 min to remove cell debris. Proteins were separated using SDS-PAGE under reducing conditions and transferred to polyvinylidene fluoride membranes. The membranes were blocked and probed with Gal-3 (Abcam, Cambridge, MA, USA), MMP-9, Fas (Abcam), Bax, bcl-xl, phospho-Akt, or total Akt antibody (Cell Signaling Technology, Inc, Danvers, MA, USA) at 4 °C overnight. Finally, the membranes were incubated with a horseradish-peroxidase conjugated secondary antibody (Jackson ImmunoResearch, West Grove, PA, USA) and developed using a chemiluminescence detection kit (Millipore). An anti-GAPDH (Cell Signaling Technology) antibody was used as a loading control. Images were captured using a BioSpectrum 600 Imaging System (Ultra-Violet Products Ltd., Cambridge, UK).

### 4.11. Terminal Deoxynucleotidyl Transferase dUTP Nick end Labeling Staining

To localize cells undergoing nuclear DNA fragmentation, terminal deoxynucleotidyl transferase dUTP nick end labeling (TUNEL) was performed using in situ apoptosis detection kit (Roche, Branchburg, NJ, USA). Cells were fixed with para-formaldehyde. After the cells had been washed with phosphate-buffered saline (PBS), the TUNEL assay was performed according to the manufacturer’s protocol with the fluorescein conjugated probe. The cells were counter stained with Hoechst to assess their nuclear morphology. The Hoechst staining was performed by incubatingthe slides for 5 min at room temperature with 1 mg/mL Hoechst (Sigma) diluted in PBS. The slides were washed and mounted for analysis under a fluorescent microscope (ZEISS, Jean, Germany).

### 4.12. Flow Cytometry

In brief, cells were fixed (4% paraformaldehyde, 15 min 4 °C) and then incubated in the presence or absence of 0.1% Triton-X100 at 4 °C for 15 min. Permeabilized cells were stained with Fas antibody, and cells stained positive were visualized with an Alexa Fluor 488 Conjugate (Life Technologies, Carlsbad, CA, USA). For control staining, rabbit antiserum to an unrelated peptide was used instead of anti-Fas. Cellular fluorescence was analyzed using a FacsCanto II cytometer (BD Biosciences, San Jose, CA, USA), and data were analyzed using FlowJo software (Tree Star, Inc. Ashland, OR, USA).

### 4.13. Statistical Analyses

SPSS software 17.0 was used for the analysis. All values are expressed as the means ± SEM. For analysis of Gal-3 level in plasma, unpaired t test was performed. Multivariate logistic regression analysis was performed to assess the association between Gal-3 with AAA presence adjusting for the potential identified confounders. In vitro experiments were replicated at least three times and were analyzed by one-way ANOVA and the Tukey post-hoc test. Statistical significance was defined as * *p* < 0.05. ** *p* < 0.001. *** *p* < 0.0001.

## 5. Conclusions

The present findings demonstrate that Gal-3 is a promising biomarker for identifying patients who are prone to AAA development. This study revealed that the beneficial effects of Gal-3 inhibition on macrophage-associated inflammation and SMC apoptosis, which might serve as a suitable alternative for therapeutic strategies in the near future.

## Figures and Tables

**Table 1:** Demographic characteristics of control subjects and AAA patients.

Total No.	CTL	AAA	*p* Value
	97	76	
Aortic diameter (mm)	20.1 ± 1.7	61.4 ± 10.8	<0.0001
Age, yr	70.5 ± 10.3	78.8 ± 8.6	0.007
Sex (male/female)	46/51	66/10	<0.001
Body weight, kg	66.3 ± 11.8	64.4 ± 12.6	0.659
Height, cm	160.4 ± 9.0	163.3 ± 7.9	0.226
Hypertension (%yes)	86.6	75.0	0.075
Smoke (%yes)	15.5	61.8	<0.001
Hypercholesterolemia (%yes)	50.5	27.6	0.003
DM (%yes)	31.9	17.1	0.034
Peripheral vascular disease(%yes)	1.0	4.0	0.321
COPD (%yes)	4.1	19.7	0.001
Medications:			
ACEi/ARB (%yes)	65.0	48.7	0.043
Statin (%yes)	42.3	25	0.024
β-blocker (%yes)	30.9	39.5	0.263
calcium channel blocker (%yes)	44.3	55.3	0.170
Aspirin (%yes)	8.2	17.1	0.101
anti-coagulants (%yes)	5.2	2.6	0.468
Antiplatelets (%yes)	6.2	31.6	<0.001

Continuous variables are presented as mean ± standard deviation and categorical variables are expressed as numbers. Abbreviations: CLT, control group; AAA, abdominal aortic aneurysm; DM, Diabetes mellitus; COPD, chronic obstruction pulmonary disease; ACEi, angiotensin-converting enzyme inhibitor; ARB, angiotensin receptor blocker. A Mann–Whitney *U*-test was used to compare continuous variables, and Fisher’s exact test (two-sided) was used to compare categorical data. *p* < 0.05.

**Table 2:** Association between plasma Gal-3 levels and clinical variables of AAA.

Variables	OR (95% CI)	*p* Value
Model 0	1.106 (1.062−1.151)	0.000
Model 1	1.026 (1.015−1.037)	0.000
Model 2	1.104 (1.057−1.152)	0.000
Model 3	1.018 (1.009−1.027)	0.000
Model 4	1.017 (1.009−1.025)	0.000

Abbreviations: CI, confidence interval; OR, odd ratio; Abbreviations: DM, Diabetes mellitus; COPD, chronic obstruction pulmonary disease; ACEi, angiotensin-converting enzyme inhibitor; ARB, angiotensin receptor blocker. Model 0: crude effect (without adjustment); Model 1: adjusted by age (> 65 years), gender; Model 2: adjusted by smoking; Model 3: adjusted by hyperlipidemia, DM, and COPD; Model 4: adjusted by use of ACEi/RBC, statins, and antiplatelet.

## Abbreviations

AAA	Abdominal Aortic Aneurysm
SMC	Smooth Muscle Cell
MMP	Matrix Metalloproteinase
CREB	cAMP-response element binding protein
NF-κB	Nuclear factor-κB
CCL5	C-C motif chemokine 5
STAT3	Signal Transducer and Activator of Transcription 3
EDTA	Ethylene-Diamine-Tetra-Acetic Acid
LPS	Lipopolysaccharide
IFN-γ	Interferon-gamma
MCP-1	Monocyte Chemoattractant Protein-1
CM	Conditioned Media
IL	Interleukin
TNF-α	Tumor Necrosis Factor-alpha
GAPDH	Glyceraldehyde 3-phosphate Dehydrogenase
TUNEL	Terminal Deoxynucleotidyl Transferase dUTP Nick End Labeling
RT-PCR	Reverse Transcription Polymerase Chain Reaction
